# The effect of concurrent reward on aversive information processing in the brain

**DOI:** 10.1016/j.neuroimage.2020.116890

**Published:** 2020-04-30

**Authors:** Andy J. Kim, Brian A. Anderson

**Affiliations:** Texas A&M University, Department of Psychological & Brain Sciences, Texas A&M Institute for Neuroscience, 4235 TAMU College Station, TX, 77843-4235, USA

**Keywords:** Reward, Punishment, fMRI, Decision-making

## Abstract

Neural networks for the processing of appetitive and aversive information, in isolation, have been well characterized. However, how the brain integrates competing signals associated with simultaneous appetitive and aversive information is less clear. In particular, it is unknown how the presence of concurrent reward modulates the processing of an aversive event throughout the brain. Here, we utilized a four-armed bandit task in an fMRI study to measure the representation of an aversive electric shock with and without the simultaneous receipt of monetary reward. Using a region of interest (ROI) approach, we first identified regions activated by the experience of aversive electric shock, and then measured how this shock-related activation is modulated by concurrent reward using independent data. Informed by prior literature and our own preliminary data, analyses focused on the dorsolateral prefrontal cortex, anterior and posterior insula, anterior cingulate cortex, and the thalamus and somatosensory cortex. We hypothesized that the neural response to punishment in these ROIs would be attenuated by the presence of concurrent reward. However, we found no evidence of concurrent reward attenuating the neural response to punishment in any ROI and also no evidence of concurrent punishment attenuating the neural response to reward in exploratory analyses. Altogether, our findings are consistent with the idea that neural networks responsible for the processing of reward and punishment signals are largely independent of one another, and that representations of overall value or utility are arrived at through the integration of separate reward and punishment signals at later stages of information processing.

## 1. Introduction

Decision-making involves the evaluation of the potential costs and benefits of different possible actions one can take, based on prior experience, that ultimately results in choice behavior (O’Doherty et al., 2017). Effective decision-makers seek to maximize rewards while minimizing aversive outcomes and thereby maximize overall utility. In experimental paradigms, decision-making tasks commonly consider the role of reward or punishment in isolation, in which the explicit goal of the task is either to maximize reward or minimize punishment (Burnett et al., 2010; Figner et al., 2009; Ilango et al., 2012; Navalpakkam et al., 2010; Paulsen et al., 2011; Singh and Khan, 2012; Stark et al., 2004; Stevens et al., 2014; Worthy et al., 2011). Even tasks that incorporate both reward and punishment, such as the Iowa Gambling Task, often utilize a design in which a given choice is either rewarded (monetary gain) or punished (monetary loss), and the goal of the task is to maximize overall rewards across multiple decisions (Bechara et al., 1994; Toplak et al., 2010). Under such conditions, a given outcome is either distinctly positive or distinctly negative, and these individual outcomes must be integrated over time in order to arrive at a representation of overall utility.

What about situations in which a given decision can have multiple consequences, some of which are positive and some of which are negative? In this sort of situation, it is necessary to balance the weight of these positive and negative outcomes in order to arrive at an integrated representation of the overall utility of a single decision. A given reward is not as desirable in situations in which it is accompanied by an aversive outcome, and a given punishment is not as strong of a deterrent in situations in which it is accompanied by a rewarding outcome. How does the brain perform the computations necessary to arrive at an understanding of such tradeoff?

One possibility is that the brain represents reward and punishment independently via distinct neural systems specialized for detecting the respective outcome, and these punishment- and reward-specific representations are only integrated into something akin to a common utility currency at a later stage of value integration (Yacubian et al., 2006). By this account, the brain network activated by punishment will be largely unaffected by reward considerations. On the other hand, the receipt of reward may affect the representation of the punishment itself and vice versa, with one outcome suppressing the representation of the other. By this second account, reward and punishment share a competitive relationship in how they are represented in the brain. Such competition could extend all the way to the sensory-discriminative aspects of aversive information processing, or be restricted to the affective and/or cognitive-evaluative aspects.

A distributed neural network involved in the processing of the sensory-discriminative, affective, and cognitive-evaluative components of aversive outcomes has been identified that includes the thalamus and somatosensory cortex (DaSilva et al., 2002; Davis et al., 1998; Wager et al., 2013), dorsolateral prefrontal cortex (dlPFC, see Seminowicz and Moayedi, 2017, for an extensive review), anterior insula (AI, Davis et al., 1998; Starr et al., 2009; Wager et al., 2013), posterior insula (PI, Davis et al., 1998; Kross et al., 2011; Wager et al., 2013), and anterior cingulate cortex (ACC, Fuchs et al., 2014; Price, 2000; Wager et al., 2013). The thalamus, somatosensory cortex, and PI have been implicated in the sensory-discriminative dimension of aversive information processing (e.g., DaSilva et al., 2002; Davis et al., 1998; Wager et al., 2013), the AI and ACC in the affective dimension (e.g., Davis et al., 1998; Fuchs et al., 2014; Price, 2000), and the dlPFC in the cognitive-evaluative dimension, endogenously mitigating the experience of pain (Seminowicz and Moayedi, 2017). The extent to which these different components of punishment processing are integrated with the representation of other outcomes (and in particular, reward) is unclear.

Some studies have utilized the approach-avoidance conflict (AAC) paradigm to identify brain regions specifically recruited during conflict (Amemori and Graybiel, 2012; Aupperle et al., 2015). Others have examined interactions between reward and punishment in neuro-economical models of valuation (Park et al., 2011) and in the anticipation of possible outcomes (Choi et al., 2014), suggesting widespread representations of expected value that integrate reward and punishment information. Talmi et al. identified the modulation of reward anticipation in the ventral striatum and rostral anterior cingulate cortex (rACC) by anticipated punishment: reward predictions were reduced when a painful compared to a neutral stimulus would accompany the reward (Talmi et al., 2009). However, to our knowledge, the effect of concurrent reward on the processing of punishment outcomes (i.e., the experience of the punishment itself) remains unexplored. In an effort to address this gap in knowledge, our focus in the present study was explicitly on the role of reward in modulating the neural representation of punishment.

In the present fMRI study, we used monetary reward and punishment in the form of electric shock to examine the modulation of punishment processing due to simultaneous presentation of reward. Monetary gains and electric shock are frequently manipulated to study reward and punishment processing in the brain, including in studies assessing the relationship between them (e.g., Choi et al., 2014; Talmi et al., 2009). Although they differ in that money is a secondary reinforcer and shock is a primary punisher, the manipulation of monetary reward still permits an opportunity to assess whether concurrent reward influences the representation of punishment more broadly. In light of prior precedent, we chose to use money as a reward and electric shock as a punishment outcome.

More specifically, to address our research question, we utilized a functional localizer approach (Poldrack, 2007) to identify regions of interest (ROIs) involved in representing punishment and probe the nature of these representations during situations of conflicting reward and punishment (presented simultaneously), punishment only, and no feedback. ROIs sensitive to the experience of punishment without concurrent reward were identified through a localizer scan. Our ROIs encompassed brain areas previously identified in punishment processing including the dorsolateral prefrontal cortex (dlPFC), anterior and posterior insula (AI/PI), anterior cingulate cortex (ACC), and the thalamus and somatosensory cortex (SSC). During the main portion of the experiment, participants performed a four-armed bandit task with pseudo-random outcome probabilities, where reward alone, punishment alone, reward and punishment simultaneously, or no outcome are equiprobable. In this sense, our behavioral paradigm amounts to a gambling task, although we included a choice element in an effort to help maintain participant engagement and render the outcomes behaviorally relevant. By equating the frequency of different outcomes, we were able to measure outcome-specific processing uncontaminated by differences in prediction errors brought about by differences in learning or behavioral strategies. We therefore limited our research question to decision-making contexts in which outcomes follow actions that participants take, which has similarly been the focus of related work in this area (Choi et al., 2014; Talmi et al., 2009).

Of interest in the present study was whether and where punishment-sensitive responses in brain will be reduced by the receipt of concurrent reward, with the experience of reward attenuating the representation of the aversive outcome. Based on the work by Talmi et al. (2009) in the context of reward prediction and work by Choi et al. (2014) in the context of outcome anticipation, we hypothesized that at least some of the aforementioned ROIs will demonstrate an attenuated response to punishment in the presence of concurrent reward. We did not have specific hypotheses concerning which regions will show this attenuated response and therefore endeavored to include a wide variety of punishment-sensitive regions in our analyses to provide a comprehensive account of the nature of reward-dependent modulation.

### Hypotheses:

Our overarching hypothesis was that the neural response to punishment will be attenuated by concurrent reward. Specifically, concerning the hemodynamic response to feedback: punishment alone > simultaneous punishment and reward. This hypothesis was tested in the following ROIs that were both determined *a priori* and supported by pilot data (see Methods), being examined independently for each ROI given that we have no *a priori* hypotheses concerning which regions are more or less likely to demonstrate the predicted relationship:

**H1a**. right dlPFC
**H1b**. left dlPFC
**H1c**. mid ACC
**H1d**. thalamus (bilateral)
**H1e**. contralateral (right) somatosensory cortex
**H1f**. left AI
**H1g**. right AI
**H1h**. left PI
**H1i**. right PI

## 2. Methods

### 2.1. Participants

At least 29 but no more than 40 participants were proposed *a priori* to be recruited from the Texas A&M University community (see Preliminary Data and Power Analysis below). 33 participants were recruited but two participants withdrew from the study prior to completing the fMRI session (see Experiment Procedure). Thus, 31 participants (16 females), between the ages of 18 and 35 inclusive (M = 22.8y, SD = 4.1y), ultimately completed the study and contributed data. All participants reported normal or corrected-to-normal visual acuity and normal color vision. All procedures were approved by the Texas A&M University Institutional Review Board and are consistent with the principles expressed in the Declaration of Helsinki. Written informed consent was obtained for each participant.

### 2.2. Preregistration of study protocol

The approved study protocol was made publicly available following in principle acceptance on the Open Science Framework (DOI:10.17605/OSF.IO/A5PXZ, https://osf.io/a5pxz/), prior to data collection (excluding the pilot data referenced below and in the approved protocol).

### 2.3. Experiment Procedure

Participants were scheduled for an initial in-lab visit for 1 h and a scan-center visit on the following day. During their initial appointment, participants came into the lab for consenting, MRI safety screening, and to practice the decision-making tasks to acquire familiarity with the different possible outcomes as well as the stimulus-response mapping. Each eligible participant completed a single 1 h fMRI session that took place the following day. During the fMRI session, participants completed 4 runs of the (main) conflict task, an anatomical scan, and a punishment functional localizer scan. Data from a functional localizer scan for regions sensitive to reward feedback were also acquired for a pilot study examining the effect of reward feedback on stimulus-specific reactivation in the visual cortex that is unrelated to the questions posed in the present study. The functional localizer scans were performed after the main task in order to avoid potentially biasing participants towards reward-seeking or punishment-avoidance; this order allows us to assess how reward modulates punishment responses when the two outcomes were only ever equiprobable in the task.

### 2.4. Apparatus

During the initial in-lab visit, all tasks were completed on a Dell OptiPlex 7040 computer (Dell, Round Rock, TX, USA) equipped with Matlab software (Mathworks, Natick, MA, USA), and Psychophysics Toolbox extensions (Brainard, 1997). Stimuli were presented on a Dell P2717H monitor. The participants viewed the monitor from a distance of approximately 70 cm in a dimly lit room. Paired electrodes (BioPac Systems, Inc., Goleta, CA, USA) were attached to the left forearm of each participant, and electric shocks were delivered through an isolated linear stimulator under the constant current setting (STMISOLA, BioPac Systems), which were controlled by custom Matlab scripts.

For the fMRI portion of the experiment, stimulus presentation was controlled by an Invivo SensaVue display system. The eye-to-screen distance was approximately 125 cm. Key responses were entered using two Cedrus Lumina two-button response pads. MRI-compatible electrodes (BioPac Systems) were attached to the left ankle of each participant, and electric shocks were delivered through an STM100C controlled by an MP160 system (BioPac Systems) triggered by custom Matlab scripts via parallel port interface.

### 2.5. Procedure

**Fig. 1.**
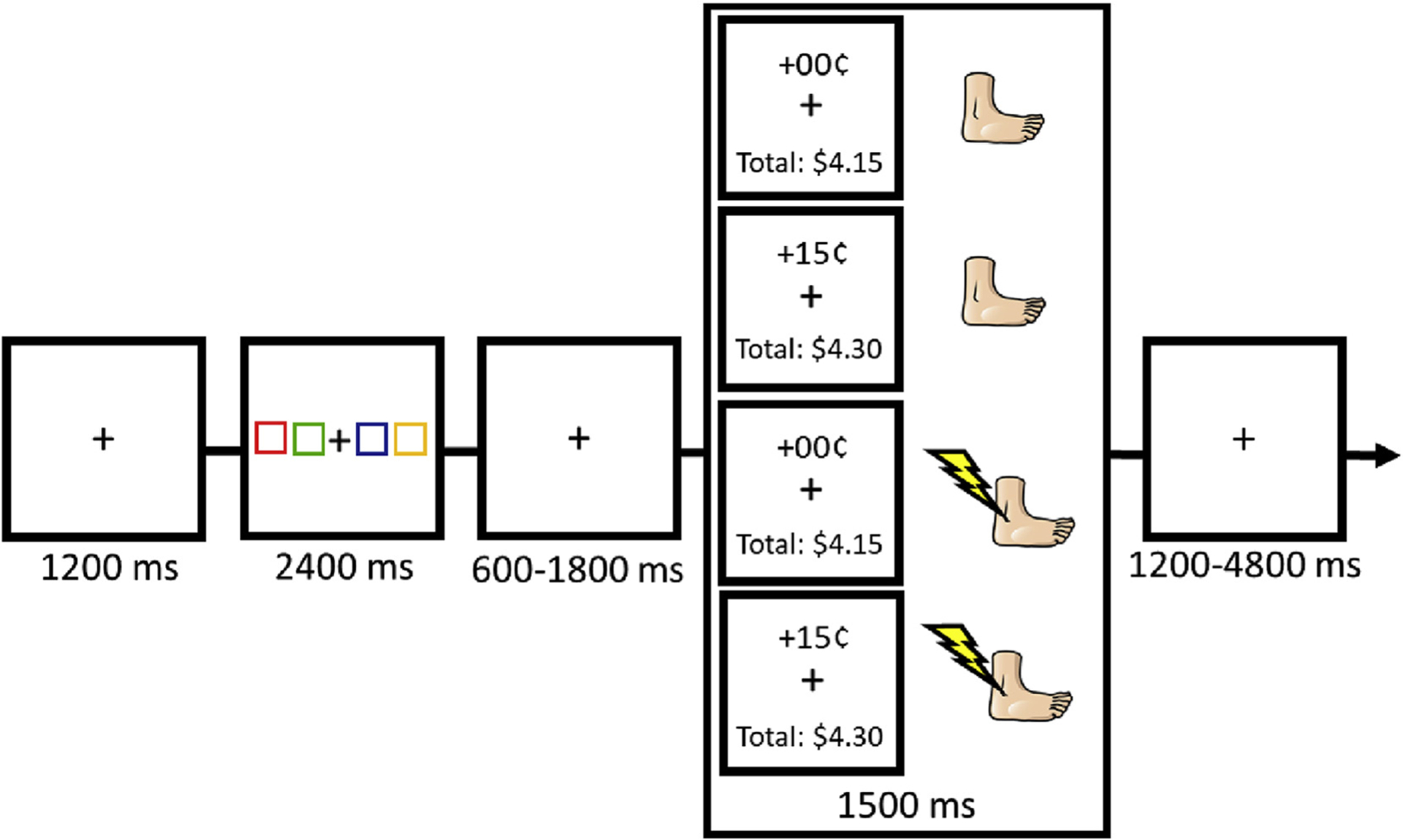
Sequence of events for a trial in the main task of the experiment. Each of the four possible outcome pairings occured equally-often in each run of the task. For the punishment localizer task, no monetary rewards are ever received, and half of all trials result in a shock outcome.

For all tasks, each trial consisted of a fixation display, choice array, an inter-stimulus-interval (ISI), a feedback display, and an inter-trial-interval (ITI). The fixation display consisted of a white fixation cross (0.8° × 0.8° visual angle) for 1200 ms (see Fig. 1). The choice array consisted of the fixation cross flanked by two boxes to the left and right (each box 3.7° × 4.8°). The two inner boxes were 4.9° center-to-center from the fixation cross and the two outer boxes were 7.4° center-to-center from the neighboring box. The color of each box was drawn from the following set without replacement {red (RGB: 255 0 0), green (RGB: 0 255 0), blue (RGB: 0 0 255), yellow (RGB: 255 255 0)}.

Prior to completing the decision-making tasks, participants underwent a shock calibration procedure to achieve a level of shock that is “unpleasant, but not painful” (Murty et al., 2012; Schmidt et al., 2015, 2017). In the task, participants were instructed to choose a colored box, but if a decision was not made fast enough a random box was chosen for them. During the in-lab portion, box choices were made using the keyboard via the “Z”, “X”, “N”, and “M” keys corresponding to location from left-to-right. During the fMRI portion, box choices were made using two dual-button response pads (middle and index finger of each hand), again corresponding to the location of the boxes from left-to-right. The choice array remained on screen for 2400 ms. Whether or not a response was logged within this 2400 ms, all four boxes disappeared and only the fixation cross remained visible during the ISI. The ISI lasted for 600, 1200, or 1800 ms (equally distributed). The feedback display was then presented for 1500 ms, which consisted of the fixation cross, the amount of monetary reward earned on the current trial (+15¢ or 00¢), and the total reward accumulated across all trials. In this way, the reward and no reward feedback differ only in the magnitude of the monetary increment indicated (+15¢ or +00¢), being equated for reading demand and number of characters (physical salience). Electric shock, if administered on that trial, was delivered simultaneously with the onset of the feedback display. Lastly, the ITI lasted for 1200, 3000, or 4800 ms (exponentially distributed, with 1200 ms occurring most frequently). The fixation cross disappeared for the last 200 ms of the ITI to indicate to the participant that the next trial was about to begin.

### 2.6. Design

The punishment localizer task consisted of one run of 64 trials while each of the 4 runs of the conflict task consisted of 32 trials. During the punishment localizer task, there was never any money earned (every trial was +00¢). To determine the ROIs, the localizer task was designed to yield punishment or no punishment an equal number of times regardless of the choices participants made. Similarly, for the conflict task, the four outcomes of reward only, punishment only, simultaneous reward and punishment, or no outcome were equiprobable regardless of the choices participants made, such that all possible outcomes were experienced with equal frequency for every participant. It is important to note that punishment only feedback was still accompanied by text indicating the (lack of) monetary increment and total earnings, such that the receipt of reward was not accompanied by increased information processing demands. The location of each color was determined randomly on each trial, and the order of outcomes was pseudorandomly determined with the constraint that each type of outcome occurred equally-often in each run of the task.

### 2.7. MRI data acquisition

Images were acquired using a Siemens 3-T MAGNETOM Verio scanner with a 32-channel head coil at the Texas A&M Institute for Preclinical Studies (TIPS), College Station, TX. High-resolution whole-brain anatomical images were acquired using a T1-weighted magnetization prepared rapid gradient echo (MPRAGE) pulse sequence [150 coronal slices, voxel size = 1 mm isotropic, repetition time (TR) = 7.9 ms, echo time (TE) = 3.65 ms, flip angle = 8°]. Whole-brain functional images were acquired using a multiband T2*-weighted echo planar imaging (EPI) pulse sequence [56 axial slices, multiband factor = 8, TR = 600 ms, TE = 29 ms, flip angle = 52°, image matrix = 96 × 96, field of view = 240 mm, slice thickness = 2.5 mm with no gap]. Each EPI pulse sequence began with dummy pulses to allow the MR signal to reach steady state and concluded with an additional 6 s blank epoch to allow for measurement of the unfolding of the blood oxygenation level dependent (BOLD) response.

### 2.8. Proposed analyses

#### 2.8.1. Preprocessing

All preprocessing was conducted using the AFNI software package (Cox, 1996). Each EPI run for each participant was motion corrected using the image following the anatomical scan as a reference. EPI images were then coregistered to the corresponding anatomical image for each participant. These images were then non-linearly warped to the Talairach brain (Talairach and Tournoux, 1988) using 3dNwarpApply to aid in identification of the ROIs. Finally, the EPI images were converted to percent signal change normalized to the mean of each run, and then spatially smoothed to a resulting 5 mm full-width half-maximum using 3dBlurToFWHM.

#### 2.8.2. Statistical analysis

All statistical analyses were performed using the AFNI software package. For the localizer task, the data was subjected to a general linear model (GLM) with regressors for (1) the presentation of the choice array and corresponding button press, (2) feedback with shock, and (3) feedback without shock, in addition to standard nuisance regressors for six degrees of head motion and drift in the scanner signal. Furthermore, images during which head motion exceeds one-half the width of a voxel, along with the image preceding and following such motion spikes, were censored from analysis. Each of the three task-related regressors were modeled using 16 finite impulse response functions (FIRs), beginning at event onset. The mean beta value for each regressor from 3 to 6 s post stimulus presentation, corresponding to the approximate peak of the response, were extracted. Analysis of the data from the main task followed this same approach, only with four different feedback regressors corresponding to the four different types of possible outcomes (punishment alone, reward alone, punishment and reward simultaneously, no reward or punishment).

**Fig. 2.**
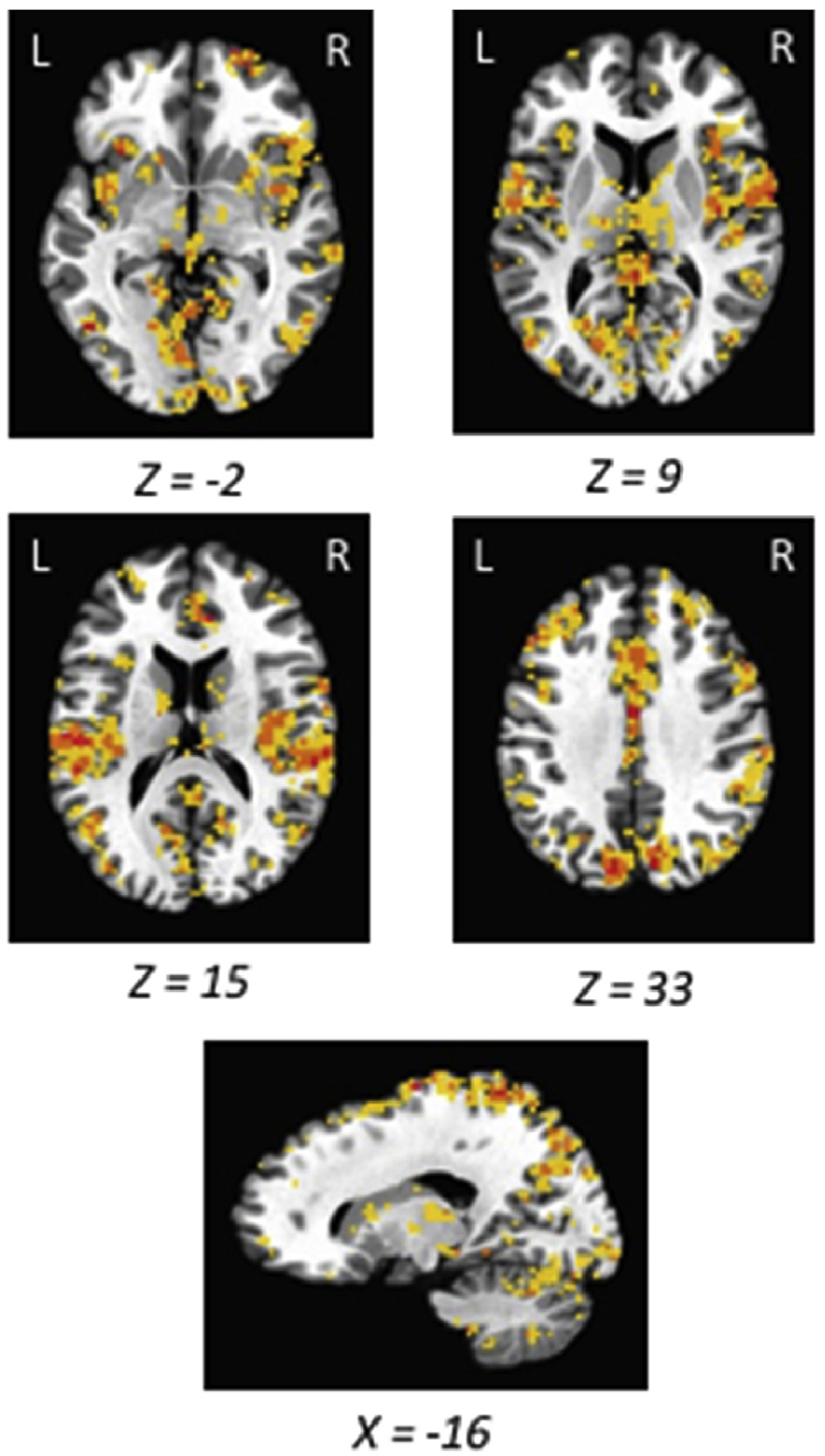
Overlapping regions of activation from the punishment localizer task used in ROI definition (pilot data, *n* = 5). Activations are overlaid on an image of the Talairach brain. Red indicates overlapping activation from all 5 participants, orange from 4 participants, and yellow from 3 participants.

To define the targeted ROIs, we contrasted the peak of the hemodynamic response (averaged over the 3–6 s time period) for feedback with and without punishment separately for each participant. Up to the 50 most significant voxels in each region (plus ties) were identified, provided that each voxelwise *p* < 0.01. To maintain consistency in ROI definition across participants, particularly for larger regions such as the dlPFC, each cluster for each ROI shared some overlap with the corresponding clusters identified in Fig. 2, with the exception of the thalamus given its small size and well-defined anatomical structure. For the main task, the peak of the response to punishment, reward + punishment, and neither outcomes were extracted from these ROIs (i.e., the mean of the beta values 3–6 s post stimulus presentation, computed separately for each voxel and then averaged over all voxels in an ROI to arrive at a mean level of activation of that ROI) and compared, with particular emphasis on the comparison of punishment with and without concurrent reward (punishment versus neither outcome served as a positive control, see below). We tested our main hypothesis separately and independently for each ROI as outlined in the Introduction, applying Bonferroni correction for multiple comparisons.

#### 2.8.3. Positive control

The appropriateness of each ROI was confirmed by contrasting punishment alone with feedback consisting of no punishment or reward, which provided a replication of the contrast used to define the region using independent data. To be included in the primary analysis (comparing punishment vs. punishment + reward), for a given ROI this contrast of punishment vs. no reward or punishment needed to be significant at *p* < 0.005. ROIs not meeting this criterion were deemed to have potentially insufficient sensitivity to detect a modulation of the punishment response by reward and excluded from the main analysis.

#### 2.8.4. Criteria for data exclusion

Data for a given participant was discarded and replaced if (a) more than 10% of all time points were censored due to motion spikes, (b) motion artifact in the anatomical image produced noticeable banding that obscures structure, (c) the participant did not make a behavioral response on at least 85% of trials, which would suggest low task engagement, and/or (d) no *a priori* ROIs were identified in the localizer scan. In the event of scanner failure or crashing of the presentation software, the run affected was repeated if available scanner time allowed; to be included in the final dataset, participants needed to complete at least 80% of trials in each of the localizer and main tasks. In the present study, no data needed to be discarded based on these criteria.

### 2.9. Preliminary Data and Power Analysis

Prior to submission as a pre-registered report, we collected data from five participants using the above-described protocol. We were able to identify bilateral AI, bilateral PI, bilateral dlPFC, thalamus, contralateral SSC, and ACC, in all five participants from the functional localizer scan, which served as the *a priori* ROIs to be used for the present study (Fig. 2) and are reflected in the *a priori* hypotheses (see Introduction).

To obtain a measure of effect size for the potentially suppressing influence of concurrent reward on the representation of punishment, we computed the primary contrast of interest (punishment alone vs. simultaneous reward and punishment) for each ROI and then collapsed across ROI for each participant to arrive at an overall average influence of concurrent reward in reducing punishment-evoked activity. The resulting effect size for this collapsed comparison was *d*_z_ = 0.863. A similar analysis using the average of the computed effect sizes for each individual region (without collapsing) yielded a slightly larger effect size estimate.

Although there are no published studies to our knowledge that have examined the role of reward in modulating the neural response to punishment, there is one study that examined a different modulator (concurrent cognitive processing) in attenuating the neural response to punishment (pain) using an ROI approach with regions that overlap with the present study (Seminowicz et al., 2004). In that study, the effect size of the attenuation effect was *d*_z_ = 0.675 or greater in each of the significant regions identified. As the lowest of all estimates obtained, we chose *d*_z_ = 0.675 for the purpose of power analysis.

A power analysis using G*Power 3 (Faul et al., 2007) with effect size *d*_z_ = 0.675, desired power β = 0.8 and α = 0.05/9 (one-tailed, experiment-wise α = 0.05, Bonferroni corrected for 9 ROIs) yielded a sample size of 29, which was the minimum sample size we proposed to collect. We proposed to collect data until (a) each *a priori* ROI was represented in 29 participants, yielding the desired minimum power in each ROI, or (b) data from 40 retained participants were collected, at which point study resources would have been exhausted. In the event that an ROI had fewer than 29 participants represented in the final sample, this ROI would be dropped from the main analysis along with its corresponding hypothesis, although the results of the contrast would still be reported as an exploratory/secondary analysis for transparency.

## 3. Results

**Fig. 3.**
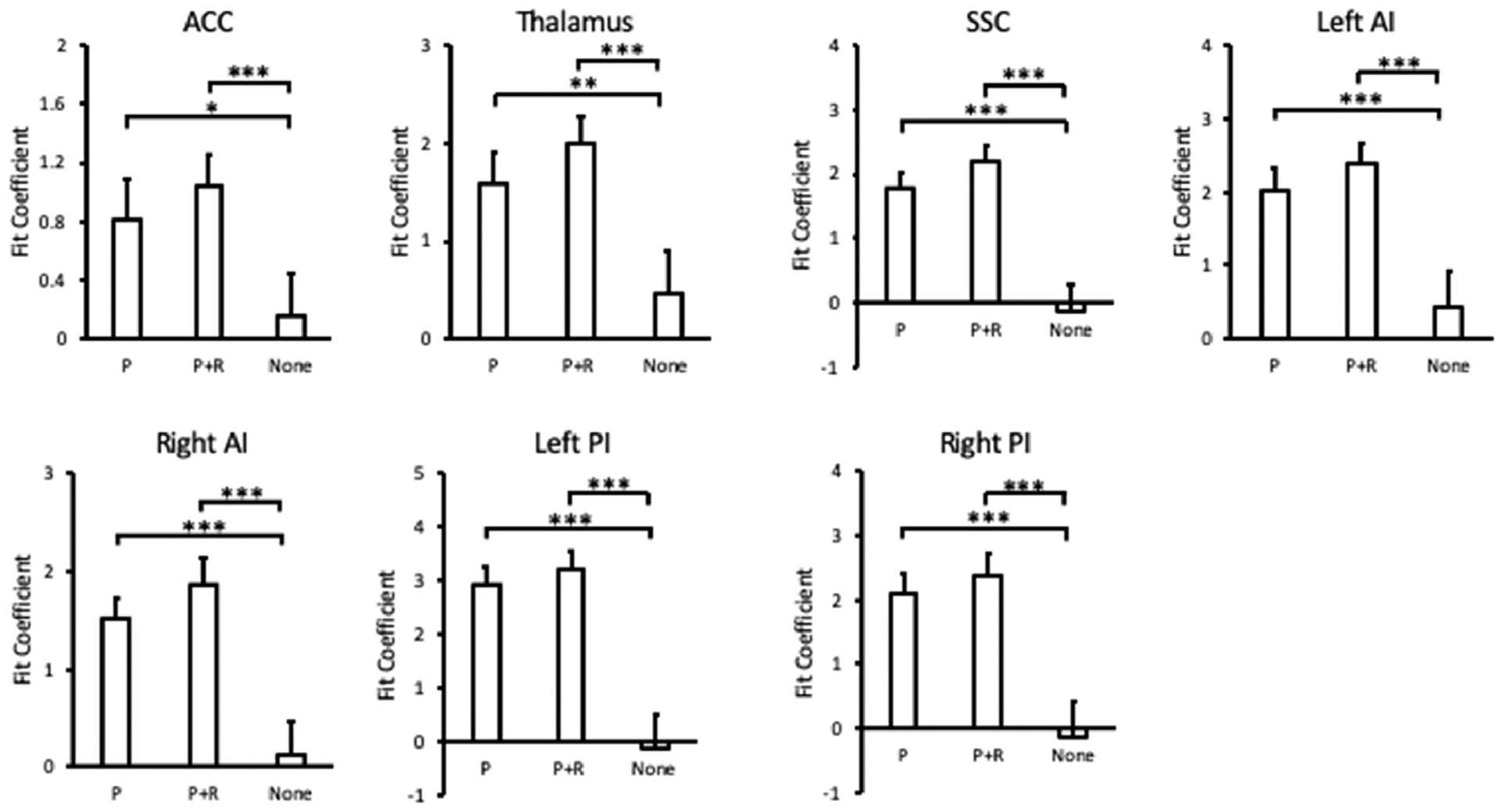
Regional activation from punishment (P), punishment and reward (P + R), and no outcome (None) across the ROIs that passed the positive control. Error bars depict within-subject confidence intervals calculated using the Cousineau method with a Morey correction. P-values indicate statistical significance following Bonferroni correction. *p < 0.05. **p < 0.01, ***p < 0.001.

All anonymized study data, digital materials/code, and the laboratory log has been made freely available on the Open Science Framework (DOI:10.17605/OSF.IO/V4HEU, https://osf.io/v4heu). After collecting data from 31 participants, all *a priori* ROIs were represented in at least 29 participants: two participants did not show significant voxel activation in the mid-ACC. We first applied our positive control assessment to each ROI by contrasting punishment feedback (P) vs. no outcome (None), which showed significant activation at the specified *p* < 0.005 level for the mid-ACC, *t*(28) = 3.05, *p* = 0.0025, thalamus, *t*(30) = 4.05, *p* = 0.00017, contralateral SSC, *t*(30) = 8.22, *p* < 0.00001, left AI, *t*(30) = 5.32, *p* < 0.0001, right AI, *t*(30) = 6.95, *p* < 0.00001, left PI, *t*(30) = 8.35, *p* < 0.00001, and right PI, *t*(30) = 7.47, *p* < 0.00001 (see Fig. 3). However, neither the left nor right DLPFC passed this positive control, *ts*(30) < 2.07, *ps* > 0.024, and were thus excluded from further analysis.

To evaluate our overarching hypothesis that the neural response to punishment will be attenuated by concurrent reward, we compared voxel activation in the aforementioned seven regions corresponding to punishment feedback (P) vs. concurrent punishment and reward feedback (P + R). In no region was a significant difference evident, with the direction of effect being opposite the direction hypothesized in each region, *p*s > 0.90 (Fig. 3). Thus, we see no evidence that reward attenuates the representation of simultaneous punishment.

### 3.1. Exploratory analyses

Given the surprising pattern of feedback responses observed above, with the direction of effect being the opposite of what was predicted, we first probed whether the apparent increase in feedback-elicited activation by concurrent punishment and reward vs. to punishment alone would have been sufficiently robust to have passed correction for multiple comparisons had our hypothesis been two-tailed. In the contralateral SSC, this was the case, *t*(30) = 4.28, *p* = 0.00018, although for the other six ROIs the difference would have been non-significant, *t*s < 2.71, *p*s > 0.011.

**Fig. 4.**
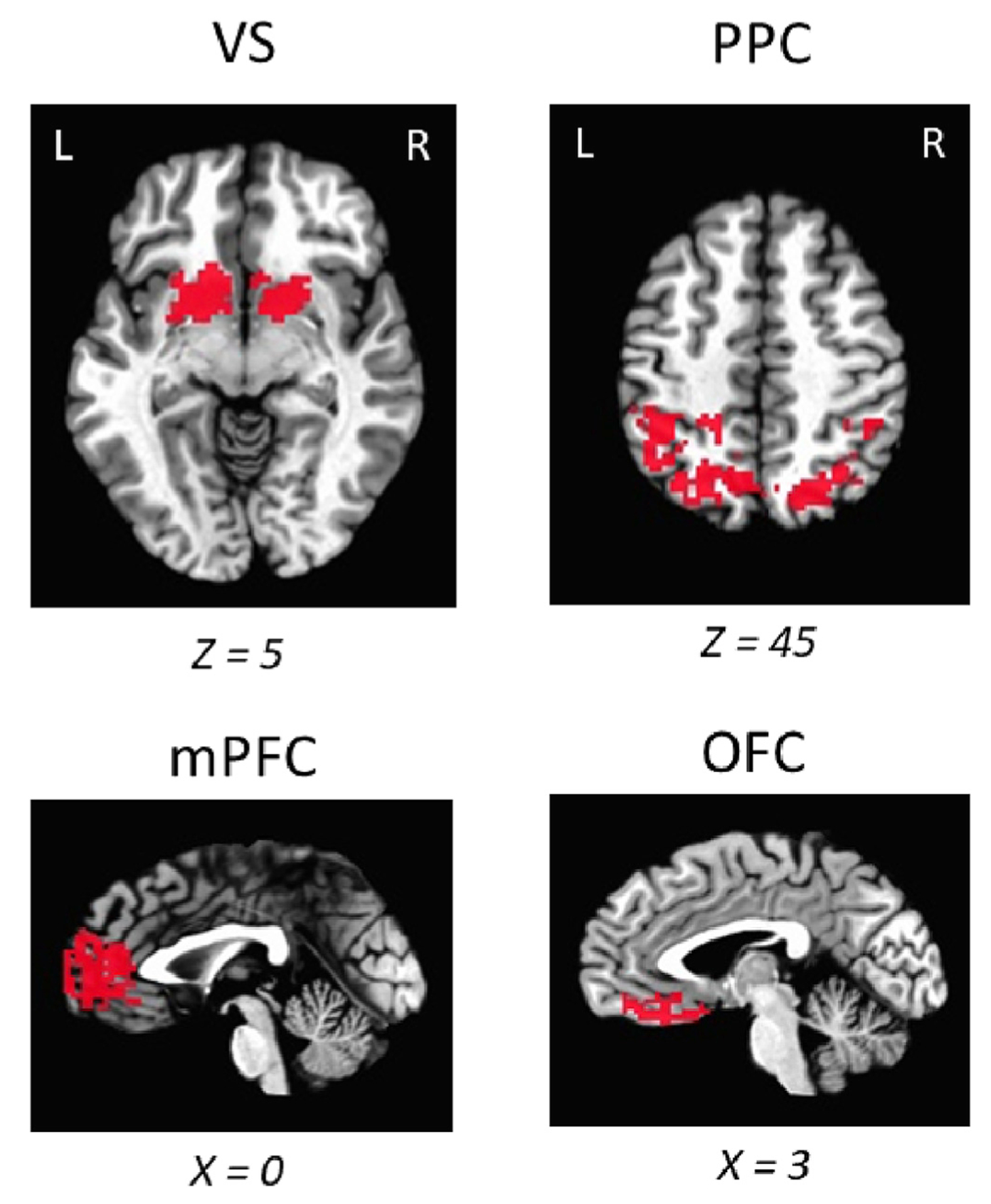
Voxels representative of each region used in reward localizer exploratory analysis. Each ROI shows the 50 most significant voxels (plus ties) for every subject combined and overlaid on an image of the Talairach brain.

**Fig. 5.**
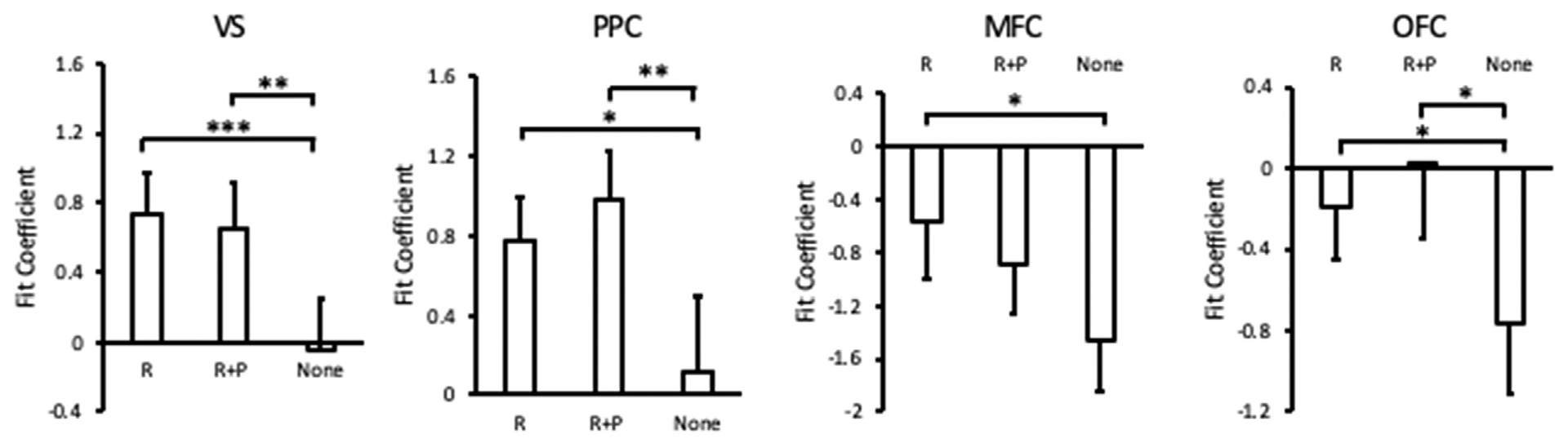
Regional activation from reward (R), reward and punishment (R + P), and no outcome (None) across the exploratory ROIs. Error bars depict within-subject confidence intervals calculated using the Cousineau method with a Morey correction. P-values indicate statistical significance following Bonferroni correction. *p < 0.05. **p < 0.01, ***p < 0.001.

In a second exploratory analysis, we looked to evaluate whether the neural response to reward would be attenuated by concurrent punishment, as suggested by a prior study focusing on reward anticipation (Talmi et al., 2009). Although it was not of primary interest, to allow us to address another research question concerning the influence of reward feedback on stimulus-specific reactivation in the visual cortex not described here, participants also completed a reward feedback localizer (see Methods), allowing us to conduct a parallel analysis concerning the manner in which concurrent punishment influences reward processing. Mirroring our analyses for the punishment localizer, but without pre-specified constraints for the boundaries of regions from pilot data, ROIs sensitive to reward feedback were identified: From data collected in the reward localizer scan of the 31 participants, we identified (A) ventral striatum (VS) in 27 participants, (B) posterior parietal cortex (PPC) in 31 participants, (C) medial prefrontal cortex (mPFC) in 30 participants, and (D) orbitofrontal cortex (OFC) in 29 participants (see Fig. 4). Again, as a positive control, we evaluated the appropriateness of each ROI by contrasting reward alone vs. feedback consisting of neither reward nor punishment (i.e., no outcome) in the main task, with a Bonferroni corrected alpha level of 0.0125 (0.05/4). Voxel activation was increased from reward feedback (R) compared to no outcome (None) in the VS, *t*(26) = 4.93, *p* = 0.00004, the PPC, *t*(30) = 2.85, *p* = 0.0079, the mPFC, *t*(29) = 3.01, *p* = 0.0054, and the OFC, *t*(28) = 4.93, *p* = 0.0059 (Fig. 5). However, we found no statistical differences in any of the ROIs when evaluating neural responses to reward feedback (R) vs. concurrent reward and punishment feedback (R + P), *t*s < 1.85, *p*s > 0.074 (Fig. 5).

**Fig. 6.**
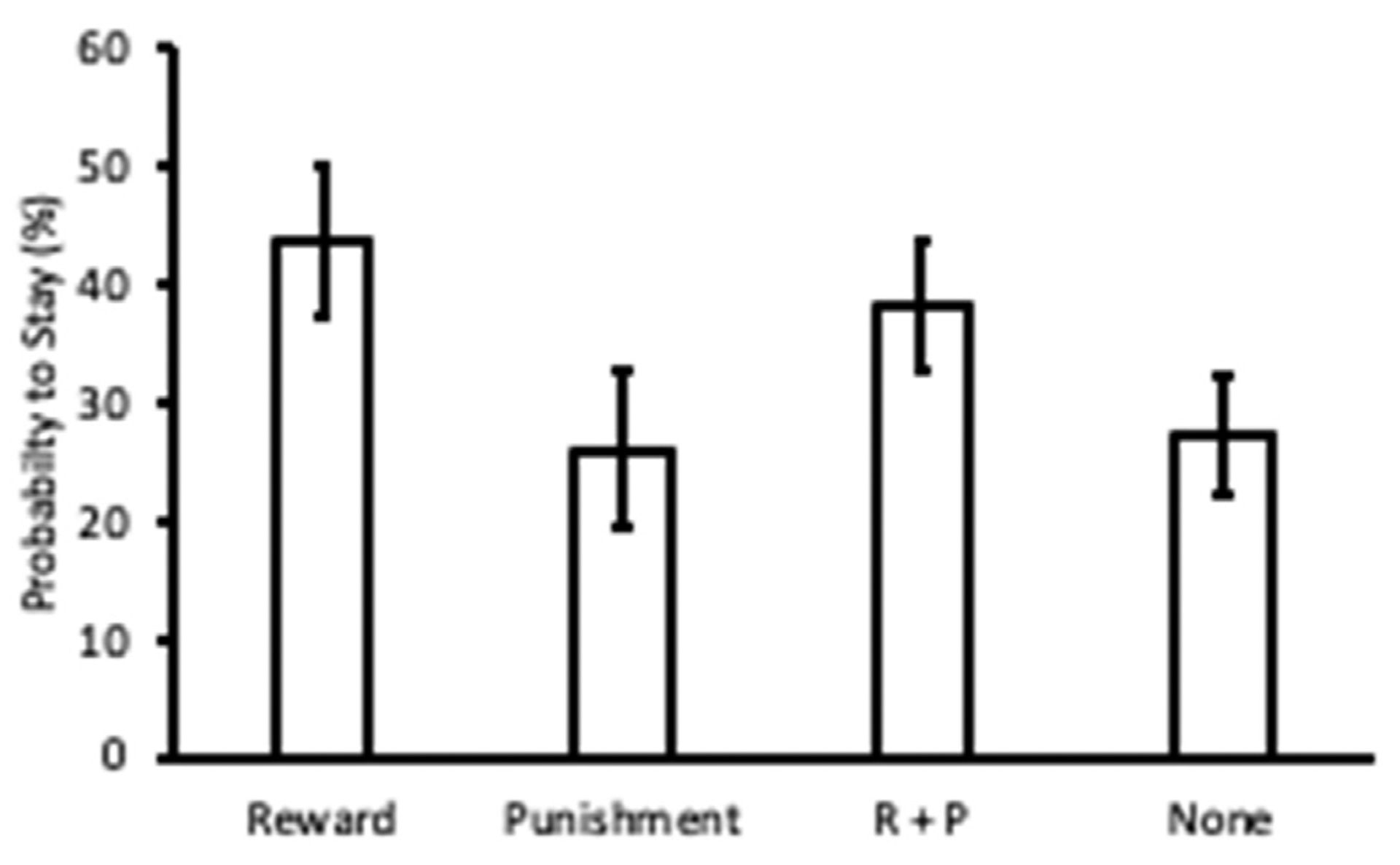
Choice behavior analysis (exploratory). Probability of choosing the same color box that was chosen on the prior trial (stay) after receiving reward, punishment, concurrent reward and punishment, or no outcome. Error bars depict within-subject confidence intervals calculated using the Cousineau method with a Morey correction.

Lastly, we looked to evaluate whether choice behavior in the task was influenced by the nature of the outcomes participants received. We conducted a within-subjects ANOVA over the probability to choose the same color box again following each type of outcome (reward, punishment, concurrent reward and punishment, no outcome) and found a significant difference depending on the outcome, *F*(1,30) = 12.73, *p* < 0.001, *η*^2^ = 0.298 (Fig. 6). To determine whether subjects were adopting a “Win-Stay-Lose-Switch” strategy, we further evaluated whether subjects were more likely to choose the same color box following receipt of reward (reward and concurrent reward + punishment vs. punishment and no feedback) and whether subjects were less likely to choose the same color box following receipt of punishment (punishment and concurrent reward + punishment vs. reward and no feedback). We found that subjects were significantly more likely to choose the same color box that they did on the prior trial following receipt of reward, *t*(30) = 3.13, *p* = 0.004, *d*_rm_ = 0.561, while the opposite was true following receipt of punishment, *t*(30) = 2.45, *p* = 0.019, *d*_rm_ = 0.443.

## 4. Discussion

Based on our neuroimaging data, we found no evidence in support of our *a priori* hypothesis that neural responses to punishment are attenuated by concurrent reward. Our ROIs for this analysis encompassed the sensory-discriminative (thalamus, somatosensory cortex, PI; e.g., DaSilva et al., 2002; Davis et al., 1998; Wager et al., 2013) and affective (AI and ACC; e.g., Davis et al., 1998; Fuchs et al., 2014; Price, 2000) dimensions of aversive information processing. The dlPFC, reflecting the cognitive-evaluative dimension (Seminowicz and Moayedi, 2017), was not reliably activated in our study, suggesting that the process of endogenously mitigating the processing of pain signals was not consistently engaged across the main and localizer tasks; perhaps the more varied outcomes in the main task disincentivized any strategic mitigation of pain signals evoked in the localizer task. Within the regions comprising the sensory-discriminative and affective dimensions of aversive information processing, we see no evidence of competition from or suppression of these signals by the concurrent processing of reward. Exploratory analyses concerning the modulation of reward processing by concurrent punishment yielded a similar pattern, with no significant difference between reward alone and reward accompanied by punishment.

Our data are instead consistent with the idea that neural networks responsible for the processing of reward and punishment signals are largely independent of one another, such that the sensory-discriminative and affective evaluation of a punisher is not itself significantly affected by potentially offsetting reward considerations. Our results fit with the idea that representations of overall value or utility are arrived at through the integration of separate reward and punishment signals at later stages of information processing (Yacubian et al., 2006) rather than through competitive interactions. In this way, our results fit better with a more modular view of feedback processing in a mixed-outcome situation.

In two prior studies of the modulatory role of punishment on reward processing, reward-related signals were found to be attenuated by the prospect of punishment (Talmi et al., 2009) and the prospect of reward and punishment were found to interact (Choi et al., 2014). However, in each of these two cases, the focus was on the *anticipation* of the respective outcomes. In this case, it appears that outcome predictions do integrate reward and punishment information, with these two considerations mutually affecting each other such that anticipated reward is attenuated by punishment. The present study suggests that the processing of the outcomes used to inform and update such predictions follows a different principle, with distinct reward and punishment representations that minimally interact.

In our exploratory analyses, we found some evidence that the processing of punishment feedback was actually accentuated by concurrent reward in the somatosensory cortex, counter to our hypothesis. In our other ROIs, such an increase was not significant when correcting for multiple comparisons. Although there is no clear explanation for this observation, one possibility is that the reward feedback increased general attention paid to the feedback event as a whole. Attention is automatically biased toward reward-related information (Anderson et al., 2011; see Anderson, 2016, 2019, for reviews), and such attentional processing can extend to other stimulus input not directly related to the reward such as contextual information (Anderson, 2015). Prior neuroimaging studies have demonstrated evidence that attention interacts within brain regions processing negative valence (e.g., Pessoa et al., 2002). Furthermore, attention has also been shown to modulate perception of aversive feedback, by both increasing or decreasing the sensation of pain (e.g., Levine et al., 1982; Bushnell et al., 1985; McCaul and Haugtvedt, 1982). In the present study, the receipt of reward may have more strongly engaged participants’ attention and thereby accentuated the sensory processing of concurrent events, including the experience of electric shock.

There are two important methodological considerations that may limit the generalizability of our study that should be considered. The first concerns the behavioral choice component of our task. In an effort to help maintain participant engagement, and in keeping with prior studies investigating the relationship between the processing of reward and punishment (Choi et al., 2014; Talmi et al., 2009), the outcomes experienced in the present study followed actions that participants took. In this context, participants were striving to optimize their outcomes, which served as teaching signals for subsequent behavior. Understanding outcome processing in such a context is important, and our data speak to this case, although it is possible that a different pattern of results might be obtained if the outcomes were completely divorced from any behavior of the participants and they simply passively received them. For example, the desire to maximize gains may have led participants to pay differential attention to trials on which a reward was received compared to trials on which no monetary reward was delivered, or vice versa if the absence of reward was more instrumental for behavioral change. This difference in the importance of the outcome to behavioral strategy may have to some degree counteracted an influence of concurrent punishment. In our exploratory analyses, we found evidence for a “Win-Stay-Lose-Switch” strategy, suggesting that participants’ choices were indeed influenced by the outcomes they received. As such, we restrict our conclusions to situations in which participants are actively striving to maximize the quality of the outcomes received.

A second methodological consideration concerns the nature of the feedback employed. Also in keeping with prior studies (Choi et al., 2014; Talmi et al., 2009), we used electric shock for punishment along with money, a secondary reinforcer, for reward. As the ROI definition of our exploratory analyses shows, the reward feedback robustly activated regions of the brain known to play an important role in reward processing (e.g., Alexander and Brown, 2011; O’Doherty, 2004; O’Doherty et al., 2001; Padoa-Schioppa and Assad, 2006; Platt and Glimcher, 1999; Samejima et al., 2005; Sugrue et al., 2004), affirming the effectiveness of the reward manipulation in recruiting the expected neural circuitry. In addition, both outcomes were behaviorally relevant (as further attested to by our exploratory analyses), and the anticipation of possible electric shock is capable of modulating signals pertaining to the anticipation of monetary reward (Choi et al., 2014; Talmi et al., 2009). With these considerations in mind, our results are inconsistent with an account by which concurrent reward is associated with a reduction in the processing of an aversive stimulus broadly. It could be argued that the reward feedback was insufficiently salient in comparison to the electric shock to modulate its processing, which is less of a consideration during the anticipation of such outcomes (Choi et al., 2014; Talmi et al., 2009). However, in our exploratory analyses, we saw no evidence for the same punishment attenuating the processing of reward feedback; this result is inconsistent with a salience-based explanation for the null effects we observed in the present study with respect to the modulatory influence of concurrent reward, provided that the putatively competitive relationship between the processing of reward and punishment is bidirectional. However, it may be the case that two more similar types of outcomes, especially two the engage the same sensory system such as pleasant and aversive physical touch, would follow a different pattern.

In conclusion, our results provide no support for the idea that the brain’s experience of punishment is attenuated by concurrent reward. Although organisms are clearly capable of integrating positive and negative feedback in the process of decision-making, including the integration of mixed or conflicting outcomes, our data do not support the idea that the integration of such mixed outcomes modulates the brain’s representation of either outcome itself. Neither the sensory-discriminative nor the affective response to aversive electric shock was attenuated by concurrent monetary gains. Our data fit better with a model in which rewarding and aversive outcomes are represented as distinct, non-interacting experiences, only to be integrated in the valuation process at later stages of information processing. In this respect, our findings place important constraints on the nature and breadth of competition between reward and punishment (see Choi et al., 2014).
